# Personal

**Published:** 1858-12

**Authors:** 


### PERSONAL.

Our very amiable cotemporary, the senior editor of the *Peninsular and Independent*, not satistfied with his recent attempt to correct our sense of propriety, seems just now much concerned about the *irascibility* of our *temper*. We are sorry to have occasioned him so much solicitude in our behalf,—but really dare not promise to do any better in future.

